# The Impact of Molar Incisor Hypomineralisation on Children and Adolescents: A Narrative Review

**DOI:** 10.3390/healthcare12030370

**Published:** 2024-02-01

**Authors:** Stephanie Shields, Tong Chen, Felicity Crombie, David J. Manton, Mihiri Silva

**Affiliations:** 1 Melbourne Dental School, Faculty of Medicine, Dentistry and Health Sciences, The University of Melbourne, Melbourne, VIC 3052, Australia; ssshiel@student.unimelb.edu.au (S.S.); d.j.manton@umcg.nl (D.J.M.); 2 Inflammatory Origins, Murdoch Children’s Research Institute, Melbourne, VIC 3052, Australia; 3 Royal Children’s Hospital Melbourne, Melbourne, VIC 3052, Australia; 4 Clinical Epidemiology and Biostatistics Unit, Murdoch Children’s Research Institute, Melbourne, VIC 3052, Australia; tong.chen@mcri.edu.au; 5 Centrum voor Tandheelkunde en Mondzorgkunde, University Medical Center Groningen, University of Groningen, 9700 AD Groningen, The Netherlands

**Keywords:** molar incisor hypomineralisation, health-related quality of life, child

## Abstract

Molar incisor hypomineralisation (MIH) is a qualitative developmental enamel defect with a prevalence of 13% worldwide. This review aims to outline the current evidence regarding the impact of MIH on children’s oral health and, more broadly, their day-to-day activities. MIH is associated with negative sequelae, including hypersensitivity, post-eruptive breakdown, the rapid development of carious lesions and poor aesthetics. Other concerns pertain to the clinical management of MIH and include difficulty in achieving local anaesthesia, increased dental fear and anxiety (DFA) and increased behaviour management problems. Oral health-related quality of life (OHRQoL) is the most standardised measure of patient impact; however, no instruments have been validated for use in MIH populations. The few existing observational studies investigating the impact of MIH on OHRQoL in children have produced conflicting results. Interventions to alleviate hypersensitivity and improve aesthetics had a positive impact on the OHRQoL of MIH-affected children. Multiple methodological issues make it difficult to measure the impact of MIH, including heterogeneity in the MIH severity classification, an overlap in the indices used to diagnose dental caries and MIH as well as the subjectivity of outcome measures for hypersensitivity and DFA.

## 1. Introduction

Molar incisor hypomineralisation (MIH) is a qualitative developmental defect of enamel characterised by demarcated opacities affecting one or more permanent first molars and often permanent incisors [1]. It is a common dental condition with a global prevalence of 13% [2]. The aetiology of MIH is not fully elucidated; however, it is purported to be multifactorial, involving systemic and environmental exposures during prenatal and perinatal periods, early childhood illnesses and genetic and epigenetic factors [3]. A higher prevalence of MIH has been reported in children with systemic diseases, including common conditions, such as fever [4], and congenital defects, such as cleft lip and palate [5]. The most frequent presentation of MIH is enamel opacities, and the severity of lesions is positively correlated with an increased number of affected teeth [6]. Negative sequelae associated with MIH include hypersensitivity, post-eruptive breakdown (PEB), rapid development of carious lesions and poor aesthetics. Affected molars experience PEB either shortly after eruption or following exposure to masticatory forces, with the application of an assessment tool in a population cohort reporting 14% of MIH-affected teeth had PEB [6]. There is an increased prevalence of incisor involvement with increasing severity of MIH [7], with anterior lesions reported to occur in 37–72% of MIH-affected children [2,8]. The potential burden of MIH for affected children may include increased risk of caries lesion development in those at increased caries risk, hypersensitivity, dental fear and anxiety, behaviour management problems and reduced oral health-related quality of life (OHRQoL). The experiences of affected children will vary according to the severity of MIH and subsequent treatment needs. It is important to recognise the impact of MIH on affected children to facilitate more patient-centred delivery of care and improve outcomes. This review therefore aims to outline the impact of MIH on children’s oral health and, more broadly, their daily activities. Electronic databases PubMed and Embase were searched using the MeSH terms “molar incisor hypomineralisation”, “health-related quality of life” and “child” and the keywords “impact”, “hypersensitivity”, “caries”, “dental fear and anxiety” and “behaviour management”.

## 2. Describing MIH Severity and Implications for Impact

Ascertaining the impact of MIH requires consideration of the broad spectrum of its clinical presentation and the varying degrees of severity. The European Association of Paediatric Dentistry (EAPD) classifies MIH lesions as mild if there are demarcated opacities without enamel breakdown, mild aesthetic concerns and sensitivity to external stimuli but not brushing [9]. Severe lesions have breakdown and associated carious lesions present, spontaneous and persistent hypersensitivity that affects function (e.g., toothbrushing and mastication) and strong aesthetic concerns that may have a socio-psychological impact [9]. The ambiguity of the definition and its arbitrary sensitivity categorisation may be why there has been a surfeit of authors proposing new severity classifications [10,11,12,13]. Alternative indices have discrepancies in the mode of severity classification, either by clinical characteristics, patient-reported sensitivity or treatment needs, as well as differences in the proposed number of severity levels in each index. This lack of consensus in the literature has produced heterogeneous data, preventing multi-centre meta-analysis of available data on MIH severity and its impact both at a tooth and patient level. Another layer of complexity in the classification of MIH severity is that MIH lesions can deteriorate with prolonged exposure to the oral environment [13], and consequently, the severity can also increase over time.

## 3. The Impact of Managing MIH

The diverse clinical presentation of MIH results in heterogeneous treatment experiences for affected children. Subsequently, it can be difficult to understand the impact of these various experiences on children’s oral health and attitude towards the dentist. Management of MIH primarily aims to prevent carious lesion development, minimize PEB, alleviate hypersensitivity and manage aesthetic concerns. Children affected by MIH can have increased treatment needs, with severely affected first permanent molars requiring 4.2 times more treatment by the age of 18 years compared to children unaffected by MIH [14]. It is reported that effective local anaesthesia is often difficult to obtain for MIH-affected teeth, which may be due to increased enamel porosity and/or exposed dentine tubules leading to pulp inflammation or because of an increased density of innervation in the pulp horn and sub-odontoblastic layer, and increased pulpal vascularity in MIH-affected molars [15,16]. Difficulty with local anaesthesia and increased hypersensitivity and treatment needs experienced by MIH-affected children may necessitate advanced behaviour management techniques. Anecdotal reports of increased utilisation of nitrous oxide relative analgesia and general anaesthesia to treat MIH-affected children with an associated increased treatment burden exist; however, this is yet to be confirmed by published research. A retrospective evaluation of children referred to a British specialist paediatric dental hospital service reported that 42.6% of MIH-affected children had general anaesthesia for treatment [17] but did not provide a comparison with unaffected children.

## 4. Negative Sequelae of MIH

### 4.1. Hypersensitivity

Hypersensitivity is the acute pain resulting from contact with thermal, tactile, chemical or osmotic stimuli that can not be attributed to a dental defect or pathology [18]. It is reported as a common issue for MIH-affected individuals [19], as the increased porosity and reduced physical properties result in hypersensitivity. All types of MIH lesions, from mild to severe, can be associated with an individual experiencing hypersensitivity, with prevalence ranging from 28 to 34.7% [20,21]. However, not all children affected by MIH report hypersensitivity [11,22]. Dentists report hypersensitivity as a frequent challenge for MIH-affected patients; however, there is limited research assessing the presentation and frequency of hypersensitivity in MIH-affected individuals [23]. The subjectivity of hypersensitivity as an outcome and discrepancies in the criteria utilised for MIH classification combined with confounding factors such as the presence of restorations and caries lesions make for an opaque comparison of the literature. Newer research utilises both self-reported and operator-quantified measurements of sensitivity, such as the Schiff Cold Air Sensitivity Scale, Visual Analogue Scale or the Wong–Baker Faces Scale, to more comprehensively capture the multi-faceted perspectives of this idiosyncratic outcome measure [20,21,22,24].

An increasing severity of MIH lesions is positively associated with an increased frequency of hypersensitivity when compared to unaffected teeth [20,21,22]. A population-based sample of 102 MIH-affected children aged 8 years demonstrated this relationship [21], although the EAPD diagnostic or severity classification was not used. In the study group, 90% of the severely MIH-affected teeth (classified via the presence of PEB exposed dentine or atypical restoration) had concomitant carious lesions, a potential confounding factor for hypersensitivity, and a similar prevalence of hypersensitivity compared to the moderately MIH-affected group (51% vs. 51.6%) was reported. An Australian study of MIH-affected children referred to specialist paediatric dental practices reported a presentation of hypersensitivity in line with this population-based research [11]. The study also found that MIH incisors were rarely associated with sensitivity, and affected molars had sensitivity to temperature eight times more frequently than sensitivity to toothbrushing. Sensitivity was the strongest predictor for an MIH-affected first permanent molar (FPM) being extracted, with a 4.3-fold [2.2, 8.5] increased risk compared to a non-sensitive FPM [11]. Restored MIH-affected teeth display similar hypersensitivity to mildly MIH-affected teeth, while defect size putatively has no influence on hypersensitivity [22]. Brown defects have a significantly higher prevalence of hypersensitivity and could be considered a predictor for hypersensitivity [20]. Severe MIH lesions with increased hypersensitivity are associated with pain during toothbrushing and sensitivity to temperature [25], which can result in suboptimal plaque control [24]. Children with severe MIH and PEB show a greater impact on dietary intake compared to mildly affected children, with reported increased difficulties with eating or drinking hot, cold or solid foods [24].

A positive correlation between age and hypersensitivity has been reported; this may be expected as the severity of MIH lesions often worsens over time [20]. In contrast, another study of 57 German children with MIH and 20 unaffected children found significantly more hypersensitivity in MIH-affected children under 8 years old when the outcome was measured by dentists via the Schiff Cold Air Sensitivity Scale [22]. This conflicted with the child-reported perception of hypersensitivity as measured by the Visual Analogue Scale [22], highlighting the influence of the diverse armamentarium used to measure this outcome. Younger MIH-affected individuals may have increased hypersensitivity because, with age, there can be increased physiological reparative dentine deposition and exposure to desensitizing agents, as well as improved awareness and ability to manage symptoms of hypersensitivity. Both studies utilised small convenience samples, and therefore, their findings cannot be extrapolated to the general population because of sampling bias and selection bias. Further research is needed to ascertain patient-related causative factors for hypersensitivity in MIH-affected individuals to enable targeted preventive therapies to reduce caries risk and improve OHRQoL.

### 4.2. Dental Caries

Dental carious lesion formation is influenced by a range of factors and involves dynamic interactions between fermentable carbohydrates, free sugars and microbial biofilm on a susceptible tooth surface with input from genetic and salivary factors [26]. Although the caries process is primarily driven by ecological dysbiosis [27] and the pathological interplay between sugar and plaque, there are multiple biologically plausible ways that MIH might influence the development of carious lesions. MIH may increase susceptibility to carious lesion development as the less mineralised, more porous enamel is less resistant to cariogenic, microbially derived challenges [28]. The decreased mineral density [29] and increased organic content of MIH enamel are associated with PEB [30] and subsequent plaque accumulation that further increases the consequences of increased caries risk [24]. The hypersensitivity that presents secondary to these physical defects can limit adequate plaque control [24] and could further increase the negative outcomes of an individual’s increased caries risk. The inferior physical properties of MIH-affected enamel have been readily translated to the assumption that MIH-affected dentitions have a higher caries risk than non-affected individuals, but this is not reflected in the literature, where there is an incongruity reported in the relationship between MIH and caries. A recent systematic review evaluated 59 papers between 2007 and 2022 and reported a significant association between MIH and caries lesions diagnosed via the WHO index (mean difference in DMFT 0.56 [0.41, 0.72]) [31]. The clinical significance of this mean difference in DMFT is difficult to interpret, but as it is below 1, it could be argued to be nominal. Jälevik and Klingberg’s cohort study demonstrated a similar increment in the total DMFT values between individuals with severe MIH and an unaffected control group in the review period from age 9 to 18 years (1.6 for MIH vs. 1.3 controls) [14], and the authors questioned the impact of MIH on caries risk. Another issue is that approximal tooth surfaces are recorded in the caries data but not scored in the MIH examination [6,32]. This method may have originated as it was considered too difficult to reliably detect MIH opacities on approximal surfaces. However, this will risk underreporting the effect of MIH on caries experience for these surfaces, particularly for severe defects where the mesial- or distal-surface hypomineralised lesion is often clearly discernible.

The conjecture surrounding the relationship between MIH and caries and the difficulties in evaluating effect size and clinical significance are primarily due to the overlap between the two indices used to measure and quantify the prevalence of MIH and carious lesions. This overlap represents a methodological challenge and reflects the complexity of clinically diagnosing between MIH lesions and carious lesions. MIH-associated atypical restorations or atypical caries lesions may also be recorded in caries charting as carious lesions or restorations. This overlap makes it difficult to understand whether MIH increases caries risk at both a surface and individual level. Perhaps the caries indices for recording MIH-associated caries or restorations should be reconsidered, and the affected surfaces with a clear MIH aetiology should be excluded or separated with a new classification code. This approach may be too simplistic, as clinically, it can be very difficult to differentiate between the two conditions, with examples being MIH defects with concomitant PEB and carious lesions or atypical restorations placed due to PEB that may also have associated caries lesions. Residual hypomineralised opacities present at restorative margins can assist with differentiation of restorations as either MIH or caries aetiology, although some cases will be indiscernible. Heitmüller and colleagues reported no increased caries risk in MIH-affected individuals when atypical restorations were excluded from the caries data [33]. However, this strategy does not consider the possibility that the aetiology of atypical restorations may involve both MIH and caries. A solution to this overlap conundrum is currently elusive; however, individual and surface-level analysis could elucidate the susceptibility of both MIH-affected tooth surfaces and individuals to dental caries.

### 4.3. Dental Fear and Anxiety

Dental fear and anxiety (DFA) are often linked with MIH, with the implication that frequent hypersensitivity, difficulty achieving local anaesthesia and increased treatment burden will trigger DFA in MIH-affected individuals. There is a paucity of research to support this claim. Strong negative feelings associated with dental treatment, which may or may not fulfil the criteria for a diagnosis of dental phobia, are classified as DFA [34]. Psychometric assessment via questionnaires is the most common method of measuring DFA, with the 15-point Children’s Fear Survey Schedule–Dental Subscale (CFSS-DS) index being the most frequently employed scale. There is a complexity to the CFSS-DS data, which is a continuous variable where authors either compare mean scores or apply a threshold anxiety score to delineate high DFA within the cohort. The mechanism of ascertaining this cut-off numerical value has no consensus in the literature, with approaches including comparison with previous investigations of children with DFA or population-based DFA prevalence studies and their published mean DFA scores. Though widely utilised and validated in children’s oral health research, the CFSS-DS is not specifically validated for use in MIH populations [35], and recently, its reliability as an instrument to investigate DFA has been questioned due to inconsistencies in geographic and patient-level outcomes [36]. Assessments via the CFSS-DS repeatedly report higher prevalence and higher levels of DFA in younger children and females, while populations in Northern Europe report lower scores [36]. This finding is unique to this index and not reported by other DFA tools, so further investigation of the validity of the CFSS-DS should, therefore, be undertaken.

The landmark papers by Jälevik and Klingberg demonstrated no increase in DFA for children with severe MIH compared to unaffected children at 9 and 18 years of age utilising the CFSS-DS index [14,37]. There was no statistically significant difference in the mean CFSS-DS scores as a continuous variable between the MIH and unaffected groups at either age point. The designs of these formative studies included only individuals with severe MIH (i.e., with PEB or atypical restorations) and aged 9 years and used parents as proxies rather than self-reported outcomes for DFA, with both having the potential to report children’s anxiety inaccurately [38]. Examining the effect of MIH on DFA is difficult as it is difficult to separate the impact of treatment, particularly in severely affected groups, as one of the most complex and multi-faceted contributions to DFA. More recently, authors reported no evidence of an association between MIH and DFA, confirming the results of Jälevik and Klingberg’s landmark study and challenging pervasive clinical dogma [39,40,41,42].

### 4.4. Behaviour Management

Behaviour management problems (BMPs) during dental treatment can potentially impact the quality of care provided and influence a child’s long-term relationship with the dental environment. Jälevik and Klingberg reported that BMPs were higher in MIH-affected individuals at both 9 and 18 years of age [14,37]. These data were collected from a retrospective analysis of treatment records for BMPs, described by authors as the dentist documenting ‘severe disruptive behaviours’ resulting in the delay of treatment or rendering treatment impossible. At the age of 9 years, children with BMPs had significantly higher levels of CFSS-DS (using an arbitrary CFS-DS cut-off for high DFA as values above the cohort’s total mean score plus one standard deviation); however, there was no association between BMPs and DFA at 18 years. Dental fear and anxiety are commonly linked with an increase in BMPs; however, the conditions are not synonymous, and there are a multitude of factors contributing to a child’s dental BMPs [34]. A seminal Swedish study from 1995 found that only 27% of children with BMPs reported DFA, and 61% of children with DFA reacted with BMPs [43]. The two papers from Jälevik and Klingberg are the sole investigations into the intersection between MIH and BMPs and the inherent subjectivity that interplays between patient and operator. These retrospective audits have the potential for inaccuracy, as examiners were not calibrated to record BMPs in a standardised manner; therefore, bias could have been introduced. A prospective study with blinded examiners calibrated to use a validated behaviour ranking tool, such as the Frankl score, and employing standardised treatment protocols could facilitate more insight into the latent BMPs present in MIH populations.

## 5. Oral Health-Related Quality of Life

Oral health-related quality of life is a subjective measure of the functional and psychosocial outcomes of oral disorders in children and the most standardised measure of patient impact [44]. This multidimensional assessment is integral to an individual’s wellbeing and general health; consequently, research aims to facilitate a more holistic understanding of the impacts of MIH and the delivery of care. Currently, there is no consensus in the literature as to whether MIH affects an individual’s OHRQoL, with some authors concluding there is no impact [45,46,47,48,49,50,51,52] and others reporting a negative association between MIH and OHRQoL [53,54,55,56,57,58,59,60,61]. These papers are cross-sectional observational studies with participants either from population-based research or convenience samples of patients referred to dental providers. The majority of studies employed instruments with the child as the informant, which is appropriate as children as young as 5 years of age can self-report OHRQoL reliably [62]. Multiple studies included responses from parents/caregivers in addition to the self-assessment, which should be regarded as complementary rather than substitutional information [63], as parents do not always perceive the child’s OHRQoL accurately [64]. This low concordance was demonstrated in three studies where parents identified a reduced OHRQoL in children with MIH, contrary to the child’s own self-assessment [47,49,53]. Although the CPQ indices have been validated for use in caries, cleft and orthodontic populations and are extensively used worldwide, they have not been validated for MIH cohorts. The authors who reported an increased impact of MIH on OHRQoL differed with respect to the outcome’s relationship with MIH lesion severity level and the location of MIH-affected teeth, as well as the affected instrument domain and whether OHRQoL was reported by the child or parent/caregiver. This heterogeneity makes a comparison of their findings challenging. These studies ambiguously attempted to answer a causal question regarding the effect of MIH on OHRQoL and have some major limitations associated with their observational design, which is largely unavoidable, and cross-sectional analyses.

Central to the discourse regarding the relationship between MIH and OHRQoL is the presence of carious lesions and previous dental treatment as mediating factors. Dental caries are well established as being associated with poorer OHRQoL in children [65] and have a higher prevalence in MIH populations. The prevalence of caries in the sample is, therefore, an important determinant. For example, a school-based study with a prevalence of caries experience of 80% (including ICDAS-II scores greater than zero) failed to show that MIH was associated with poor OHRQoL [49]. Samples of older children will have an increased concentration of confounding variables as they are more likely to have had previous dental treatment, and the severity of MIH may progress with age. Samples of children younger than the EAPD-recommended age of 8 years may have fewer severely affected teeth or participants whose first permanent molars have not all erupted [54]. However, the seemingly logical assumption of an association between increasing MIH severity and poorer OHRQoL is not supported by the current literature, with multiple reports of no effect of severe MIH on OHRQoL [47,51,53]. It may be similarly challenging to isolate the impact of MIH on OHRQoL in individuals with concomitant medical conditions, such as cleft lip and palate, and therefore, existing studies have limited their eligibility criteria to healthy children.

### OHRQoL in MIH Interventional Studies

Multiple studies have demonstrated that effective treatment of MIH-affected teeth can improve children’s OHRQoL. Hasmun and colleagues investigated the effect of minimally invasive interventions for opacities on anterior teeth on participants’ OHRQoL and reported an improvement in OHRQoL 6 months after the aesthetic treatments [66]. Another German and Austrian study of 38 children with severely hypersensitive MIH-affected molars reported both a decrease in hypersensitivity and OHRQoL scores after placement of fissure sealants [67]. Dental treatment under general anaesthesia to manage caries and MIH can also improve children’s OHRQoL, as demonstrated by a Swedish study of 132 participants [68]. These papers provide valuable insights into the experiences of MIH-affected children undergoing treatment; however, none have a control group because of ethical or logistical concerns and are therefore limited in their ability to estimate predictive or causal effects.

## 6. Implications for Future Research

This narrative review aims to expand our understanding of the impact of MIH on children by augmenting knowledge obtained from systematic reviews and clinical research, clarifying common misconceptions and identifying methodological barriers to answering pertinent questions. Narrative reviews have the unique capacity for a multilayered evaluation of a topic and its relevant literature, as well as the interpretation and application of that evidence, and are an integral parallel to more prescriptive research, such as systematic reviews [69]. The commonly cited limitation of a narrative review, the lack of objective selection of evidence, is valid but may simultaneously be a major strength if the inclusion and interpretation of the evidence are rigorously conducted within a well-defined scope [69].

Future studies investigating the impact of MIH should consider the following recommendations in their study design to reduce potential bias. Authors examining hypersensitivity should employ a diverse armamentarium with both self-reported and valid and reproducible operator-quantified measurements to evaluate this subjective outcome comprehensively, reduce measurement error bias and allow comparability. Future investigations of BMPs will elucidate the impact of MIH more accurately if they are prospective in design and use a validated behaviour ranking tool with calibrated, blinded examiners. Children should report OHRQoL as there is an incongruity between self and parent/caregiver assessment [64]. The indices utilised to measure OHRQoL require validation, or alternatively, a new OHRQoL instrument could be constructed that is targeted specifically at MIH populations to capture the idiosyncratic experiences of affected children. Interventional studies assessing the impact of treatment on OHRQoL need to include a control group in their design that provides a baseline to compare with the intervention group. This comparison is crucial for establishing causality. If this is not ethical or feasible, researchers could compare their results with longitudinal observational OHRQoL data recorded in similar populations, of which there is currently a scarcity of previous publications. This may provide further insights regarding latent MIH lesion progression, which could either deteriorate with masticatory exposure or carious lesion development or conversely improve with enamel mineral gain, reparative dentine or behaviour modification.

Studies exploring DFA should focus on the child’s perspective rather than using parents as proxies, as parents often overestimate their child’s DFA [38]. The most commonly implemented tool for measuring DFA is the CFSS-DS index, which requires further scrutiny due to unique discrepancies in prevalence values reported between different ages, sexes and geographic locations [36]. There is currently no instrument validated to measure DFA in MIH-affected children, and if available, this would increase the accuracy of outcome measurements. The investigation into the association between DFA and MIH highlights the difficulty in applying data from population-based studies to individuals as they present in clinical settings. Although the current literature suggests that MIH does not cause DFA, it is possible that children could have DFA that has been triggered by severe MIH and its associated treatment. There are multiple factors that may contribute to DFA, including the temperament of the individual, the attitude of parent/caregiver, parenting style and previous negative dental experiences [70]. It may be that traumatic previous dental experiences may only have a small effect on DFA, and subsequently. MIH is only rarely the underlying source of this experience. However, it is plausible and should be considered in future research.

Ascertaining a consensus for the MIH severity index is a priority as the assortment of severity classifications and subsequent heterogeneous data prevent multi-centre comparison and analysis of key MIH outcome measures. The diagnostic overlap between the dental caries and MIH scoring criteria could be addressed by the introduction of a new code in the caries index to separate surfaces with a clear MIH aetiology. It may be impossible to definitively distinguish between MIH and caries in all clinical scenarios; however, this new code may improve examiner awareness of this diagnostic challenge and result in more accurate scoring. Including the approximal surfaces in the MIH measurement index and scoring them as affected when the MIH-affected mesial or distal surface is clearly discernible will also improve accuracy. These methodological issues may be beyond the scope of individual authors, and guidance from leading professional bodies such as the EAPD, the International Association of Paediatric Dentistry and the American Association of Paediatric Dentistry would be welcome. These recommendations, if implemented, would improve our understanding of the impact of MIH and provide further insight into MIH more broadly.

## 7. Conclusions

Children are impacted by MIH, with evidence of increased experience of hypersensitivity, associated carious lesions, dental treatment and behaviour management problems. Children with MIH do not have higher DFA. The effect of MIH on OHRQoL is unclear; however, effective management of MIH can improve a child’s OHRQoL. Clarification of the severity classification and robust longitudinal studies are required to elucidate the impact of MIH on OHRQoL.

## Data Availability

Data are contained within the article.
